# The association between end-tidal carbon dioxide and arterial partial pressure of carbon dioxide after cardiopulmonary bypass pumping in cyanotic children

**DOI:** 10.34172/jcvtr.2021.49

**Published:** 2021-11-21

**Authors:** Behrang Nooralishahi, Rozhin Faroughi, Hooman Naghashian, Ashkan Taghizadeh, Mohammadjavad Mehrabanian, Mehdi Dehghani Firoozabadi

**Affiliations:** ^1^Tehran Heart Center, Tehran University of Medical Sciences, Tehran, Iran; ^2^School of Medicine, Tehran University of Medical Sciences, Tehran, Iran; ^3^Children’s Medical Center Hospital, Tehran University of Medical Sciences, Tehran, Iran

**Keywords:** End-Tidal Carbon Dioxide, Carbon Dioxide Pressure, Cyanosis, Children

## Abstract

***Introduction:*** Evidence suggests the high capability of non-invasive assessment of the End-tidal carbondioxide (ETCO2) in predicting changes in arterial carbon dioxide pressure (PCO2) following major surgeries in children. We aimed to compare EtCO2 values measured by capnography with mainstream device and EtCO2 values assessed by arterial blood gas analysis before and after cardiopulmonary bypass pumping in cyanotic children.

***Methods:*** This cross-sectional study was performed on 32 children aged less than 12 years with ASA II suffering cyanotic heart diseases and undergoing elective cardiopulmonary bypass pumping. Arterial blood sample was prepared through arterial line before and after pumping and arterial blood gas (ABG)was analyzed. Simultaneously, the value of EtCO2 was measured by capnography with mainstream device.

***Results:*** A significant direct relationship was found between the changes in ETCO2 and arterialPCO2 (r = 0.529, *P* = 0.029) postoperatively. According to significant linear association between postoperative change in ETCO2 and arterial PCO2, we revealed a new linear formula between the two indices: ΔPCO2 = 0.89× ETCO2-0.54. The association between arterial PCO2 and ETCO2 remained significant adjusted for gender, age, and body weight.

***Conclusion:*** the value of ETCO2 can reliability estimate postoperative changes in arterial PCO2 in cyanotic children undergoing cardiopulmonary bypass pumping.

## Introduction

The partial pressure of arterial carbon dioxide (PaCo2) is one of the effective and determining factors of blood pH, so its changes can cause many problems for critically ill patients. Because the likelihood of these changes during anesthesia is very high and it is not possible to continuously monitor PaCo2 directly, so during anesthesia, End-tidal CO2 (EtCO2) monitoring is used to estimate PaCo2, which is one of the standard monitoring methods during anesthesia and is often used as a non-invasive method for patients under anesthesia as well as in recovery and intensive care units.^1^ Capnography as a tool for measuring EtCO2 has become more common in recent years. Continuous measurement of EtCO2 is one of the methods used in the operating room for evaluation of hemodynamic state during general anesthesia and also in intubated patients. But it can even be a non-invasive, fast and reliable method for predicting PaCo2 in non-intubated patients.^2,3^ This measurement makes it possible to estimate arterial PaCo2 without the need for arterial blood sampling. If there is a constant relationship between PaCo2 and EtCO2, this method is reliable and there is no need for frequent arterial blood extraction. Using this method can provide necessary information about the patient’s respiratory status quantitatively with high reliability. Therefore, in any type of monitor, the relationship between measured EtCO2 and arterial blood PaCO2 is an important and significant point. The difference between EtCO2 and PaCO2 represents the pulmonary dead space. Acute increase in dead space, such as in pulmonary embolism, widens this gap. This criterion also indicates the presence of shunting. In some studies, the association between EtCO2 and PaCO2 has been established, but the accuracy of this method is still debated.^4-7^ In a study comparing the relationship between EtCO2 and PaCO2 in the prone and supine positions, it was found that under normal circumstances, EtCO2 is a useful tool for monitoring PaCO2, but its accuracy may decrease when using different surgical techniques and in different positions.^8^

Overall, measuring and monitoring EtCO2 is an important aspect of critical patient care. While EtCO2 monitoring was initially used by physicians to verify the location of endotracheal tubes and mechanical ventilation of patients in the emergency department, today, it is increasingly used for purposes such as monitoring the quality of cardiac resuscitation and assessing the causes of bronchospasm.^9^ In addition, studies have been performed to measure EtCO2 to predict relative PaCo2 or blood bicarbonate levels.^10^ Based on the gas sampling method, infrared monitoring of PaCO2 is divided into two mainstream and sidestream methods. Sidestream capnography depends on the amount of gas removed from where the endotracheal tube is attached to the anesthesia machine. If the gas extraction rate is higher than the exhaust gas, the sample is mixed with fresh gas and the measurement accuracy will not be correct.^11^ In the Mainstream type, because the capnometer is placed directly on the endotracheal tube, the reaction time is faster and the probability of error is low, but there is a possibility of the endotracheal tube closing due to the weight of the sensor.^12^ Sidestream capnographs are highly dependent on the amount of gas that separates from the main breath system and reaches the monitor.^13^ In the present study, mainstream devices were used. In the present study, we aimed to compare EtCO2 values measured by capnography with mainstream device and PaCO2 values assessed by arterial blood gas analysis before and after cardiopulmonary bypass pump in cyanotic children. In fact, we attempted to determine the accuracy of measuring EtCO2 by direct capnography and the effect of pumping on its measurement in cardiac cyanotic children.

## Materials and Methods

This cross-sectional study was performed on 32 children aged less than 12 years with ASA II suffering cardiac cyanotic disorders and undergoing elective surgical correction at Pediatric Medical Center in Tehran between January and March 2019. All subjects were operated on by a single team and also had the same anesthesia procedure. In this regard, reluctance of the child’s parents to cooperate in the project, recent colds, airway abnormalities, difficult intubation, high arterial pulmonary pressure, and emergency surgery were considered as the exclusion criteria. The study protocol was ethically approved by the ethical committee at Tehran University of Medical Sciences. The basis for patients’ choice is heart surgery, which requires that an arterial blood sample be taken from the patient during the operation, and from this point of view, there is no moral problem in taking an arterial blood sample.

After standard monitoring and induction of anesthesia which were the same in all patients, after initial premedication with fentanyl (2 μg/kg), induction of anesthesia was scheduled with fentanyl (5 μg/kg) plus midazolam (0.05 mg/kg) and cisatracurium (0.15 mg/kg) and achieving sufficient depth of anesthesia, tracheal intubation was performed through the nose and patients were connected to a ventilator. Immediately after intubation, the arterial line was inserted using 20 or 22 gauge arterial catheters from the radial artery of the patient’s right hand. Maintenance of anesthesia was performed with isoflurane (1.5%) and oxygen (100%). The dose of fentanyl was repeated at intervals of one hour. Arterial blood sample was prepared through arterial line before and after cardiopulmonary bypass pumping (CPB) and arterial blood gas (ABG) was analyzed by the GEM Premier 3000 machine. Simultaneously, the value of EtCO2 was measured by capnography with mainstream device. Such measurements were done both before and after CPB. Hemodynamic parameters including systolic and diastolic blood pressure, heart rate and arterial oxygen saturation were also assessed before and after cardiopulmonary bypass.

For statistical analysis, results were presented as mean ± standard deviation (SD) for quantitative variables and were summarized by frequency (percentage) for categorical variables. The values of study parameters before and after the procedure were compared using the Paired t test or Wilcoxon test. The agreement between the values of EtCO2 assessed by capnography and PaCO2 measured by arterial blood gas analysis was also examined by the Kappa agreement test. For the statistical analysis, the statistical software SPSS version 23.0 for windows (IBM, Armonk, New York) was used.

## Results

Overall, 32 children (14 male and 18 female) were included into the study. The age of participants ranged from 6 days to 12 years that 31.2% were neonates, 43.8% were infants, and 25% aged higher than 2 years. The mean weight of the patients was also 7.98 ± 6.22kg. Due to non-parametric distribution of weight in the study subjects, the median of this index was determined as 8.0 kg (first quartile = 6.25, third quartile = 9.75).

**Figure 1:**
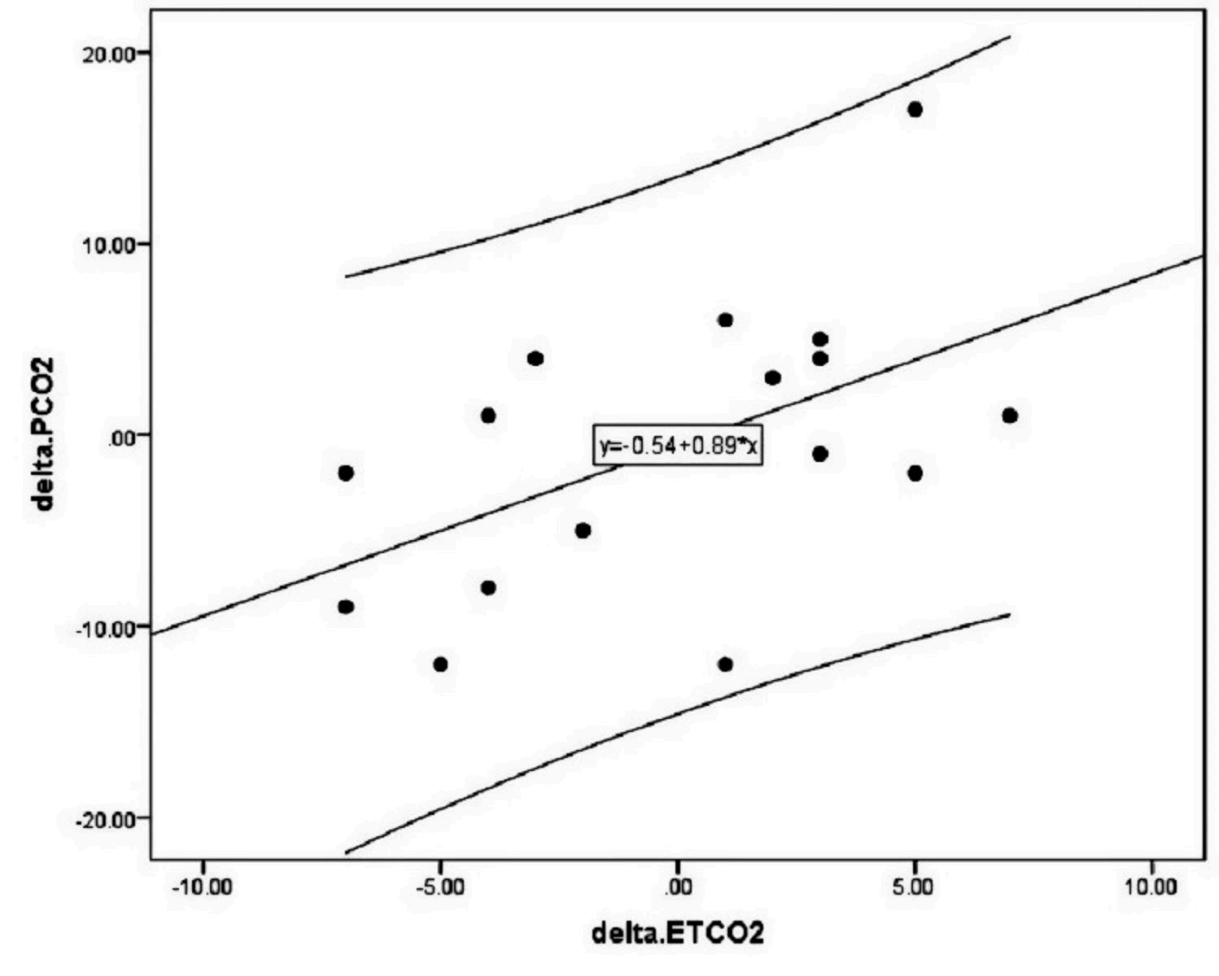
The linear association between ETCO_2_ and arterial PCO_2_

Table 1 summarizes the value of study parameters before and after procedure. Of all study parameters assessed postoperatively, the increase in systolic and diastolic blood pressures, the increase in arterial oxygen saturation despite the decrease in FiO2 were significant after procedure when compared to before. The results related to association of the postoperative change in ETCO2 and changing other study parameters (Table 2) showed only a significant direct relationship between ETCO2 and arterial PCO2 (r = 0.529, p = 0.029). According to significant linear association between postoperative change in ETCO2 and arterial PCO2, we revealed the following linear formula between the two indices: ΔPCO2 = 0.89× ETCO2-0.54. In this regard, for each unit of increase in ETCO2, the amount of change in arterial PCO2 will be equal to 0.35 units (Figure 1). Using the multivariable linear regression model with the presence of baseline variables including gender, age and body weight (Table 3), the association between arterial PCO2 and ETCO2 remained significant adjusted for baseline parameters.

**Table 1:** The value of study parameters before and after procedure

**Parameter**	**Before procedure**	**After procedure**	***P*** ** value**
Systolic blood pressure, mm Hg	76.71 ± 15.73	84.50 ± 12.00	0.031
Diastolic blood pressure, mm Hg	44.29 ± 10.89	50.35 ± 11.75	0.050
PEEP	3.00 ± 0.01	3.31 ± 0.70	0.096
Arterial pH	7.34 ± 0.08	7.36 ± 0.05	0.336
Arterial HCO_3_, meq/L	22.87 ± 3.93	23.90 ± 3.92	0.190
Arterial PO_2_	60.19 ± 27.37	128.50 ± 89.32	0.008
Arterial PCO_2_	42.29 ± 7.28	41.65 ± 6.69	0.720
ETCO2	32.12 ± 5.02	32.00 ± 3.82	0.912
FiO_2_	76.75 ± 6.93	68.44 ± 10.18	0.019
Heart rate, /minute	127.88 ± 24.31	131.12 ± 20.26	0.354

ETCO_2_: End-Tidal Carbon Dioxide; FiO_2_: Fraction of Inspired Oxygen; HCO_3_: Bicarbonate; PCO_2_: Partial Pressure of Carbon Dioxide; PEEP: Positive End Expiratory Pressure;

**Table 2:** The association between the change in ETCO_2_ parameter and change in other study indices

**Parameter**	**R coefficient**	***P*** ** value**
Systolic blood pressure, mm Hg	0.315	0.217
Diastolic blood pressure, mm Hg	0.146	0.577
PEEP	-0.410	0.114
Arterial pH	-0.239	0.373
Arterial HCO_3_, meq/L	0.221	0.411
Arterial PO_2_	0.015	0.955
Arterial PCO_2_	0.529	0.029
FiO_2_	0.447	0.083
Heart rate, /minute	0.017	0.950

ETCO_2_: End-Tidal Carbon Dioxide; FiO_2_: Fraction of Inspired Oxygen; HCO_3_: Bicarbonate; PCO_2_: Partial Pressure of Carbon Dioxide; PEEP: Positive End Expiratory Pressure;

**Table 3:** The association between ETCO2 and PaCO2 values adjusted for baseline parameters

**Item**	**Unstandardized Coefficients**		**T score**	***P*** ** value**
	**Beta**	**Standard Error**		
Change in ETCO_2_	0.911	1.447	2.040	0.044
Male gender	2.641	3.527	0.749	0.468
Age	0.001	0.004	-0.081	0.937
Weight	0.033	1.728	0.046	0.964

ETCO_2_: End-Tidal Carbon Dioxide

## Discussion

The change in both arterial PCO2 and ETCO2 parameters were not significant after cardiopulmonary bypass pumping in cyanotic children remained statistically insignificant and only hemodynamic indices including systolic and diastolic blood pressures as well as arterial oxygen saturation significantly changed that would be related to anesthesia and also procedural-related changes. However, we revealed a direct strong association between the changes in arterial PCO2 and ETCO2 indices after surgery indicating high value of assessing the changes in ETCO2 in predicting postoperative changes in arterial PCO2. The assessment of arterial PCO2 is very important to predicting critical postsurgical outcome especially hemodynamic instability that can be closely linked to patients’ poor outcome. In this regard and aided by the found linear association between PCO2 and ETCO2 parameters, we will be able to easily and noninvasively predict abnormal arterial PCO2 occurs after major surgeries.

Some other similar studies attempted to assess the link between arterial PCO2 and ETCO2 in both children and adults especially following major surgeries, however some believe that ETCO2 might underestimate the value of PCO2 in children under anesthesia. In a recent study by Arvind Chandrakantan et al,^14^ ETCO2 underestimated venous PCO2 values in neonates and infants under general anesthesia, but they introduced transcutaneous CO2 (tcPCO2) as a valuable parameters to closely approximate venous blood gas values. Wahba et al^15^ also showed that the changes in ETCO2 did not accurately indicate the direction and extent of the change in arterial PCO2. However, similar to our results, some studies emphasized the value of ETCO2 in predicting the change in hemodynamic changes following major surgeries particularly cardiac surgeries. As similarly shown by Donati et al,^16^ ETCO2 recordings were reliable, and can help in maintaining normocarbia during the short but unstable period associated with rewarming following cardiac surgery. Shibutani et al^17^ also revealed that decreases in ETCO2 quantitatively reflected the decreases in CO2 elimination. Burrows^18^ also indicated that PETCO2 correlated closely with the arterial PCO2 in the normal and acyanotic groups. Finally, Bissonnette et al^19^ showed that single breath ETCO2 measurements from the proximal end of the endotracheal tube accurately estimate the arterial PCO2 in infants and children.

As another important finding in the present study was that the value of ETCO2 measured was affected by gender, age or body weight of children. However it was shown that the value of this parameter might be affect by both parameters of patients’ age as well as the site of measuring ETCO2. As the first point, it was shown that ETCO2 monitoring may not be reliable in older ages especially in adults, but it is contrarily valuable in children.^19^ Moreover, ETCO2 measurements obtained from the proximal end of the endotracheal tube could not accurately predict arterial PCO2 values in infants and children, however proximal measurements of ETCO2 can accurately estimate arterial PCO2.^20^ Thus, measuring ETCO2 obtained from proximal zone of tube can accurately estimate postoperative PCO2 among children undergoing cardiac procedures.

## Conclusion

Despite significant differences between ETCO2 and arterial CO2 in anesthetized cyanotic children before CPB, these measures become more relevant post CPB and after surgical correction; probably due to enhanced pulmonary blood circulation.

## Acknowledgements

There are no acknowledgements to be mentioned in this study.

## Competing Interest

There is no competing interest to be mentioned in this study.

## Ethical Approval

This study was approved by the ethical committee of Tehran University of Medical sciences with approval I.D. IR.TUMS.CHMC.REC.1398.043

## Funding

There are no funding or financial interests in this study
